# The Response, Outcome and Toxicity of Aggressive Palliative Thoracic Radiotherapy for Metastatic Non-Small Cell Lung Cancer Patients with Controlled Extrathoracic Diseases

**DOI:** 10.1371/journal.pone.0145936

**Published:** 2015-12-31

**Authors:** Yun Chiang, James Chih-Hsin Yang, Feng-Ming Hsu, Yu-Hsuan Chen, Jin-Yuan Shih, Zhong-Zhe Lin, Keng-Hsueh Lan, Ann-Lii Cheng, Sung-Hsin Kuo

**Affiliations:** 1 Division of Radiation Oncology, Department of Oncology, National Taiwan University Hospital, Taipei, Taiwan; 2 Department of Internal Medicine, National Taiwan University Hospital, Taipei, Taiwan; 3 Cancer Research Center, National Taiwan University College of Medicine and National Taiwan University Cancer Center, Taipei, Taiwan; 4 Graduate Institute of Oncology, National Taiwan University College of Medicine and National Taiwan University Cancer Center, Taipei, Taiwan; Taipei Medical University, TAIWAN

## Abstract

**Background and Purpose:**

For metastatic non-small cell lung cancer (NSCLC) patients with controlled extrathoracic disease after systemic treatment, stable or progressive primary lung lesions may cause respiratory symptoms and increase comorbidities. In the present study, we sought to investigate whether aggressive palliative thoracic radiotherapy (RT) can enhance local control and improve the survival for this subgroup of patients.

**Materials and Methods:**

Between March 2006 and December 2014, 56 patients with metastatic NSCLC who had responsive or stable extrathoracic diseases after chemotherapy and/or molecular targets, and received thoracic RT for stable and progressive primary lung lesions were included. RT with a median dose of 55 Gy (range, 40–62 Gy) was administered in 1.8–2.5 Gy fractions to primary lung tumor and regional mediastinal lymph nodes using modern RT technique. Overall survival (OS) from diagnosis, and locoregional progression-free survival (LRPFS), and survival calculated from radiotherapy (OS-RT) were estimated using the Kaplan-Meier method.

**Results:**

There were 37 men and 19 women with a median age of 60 years at diagnosis. The median interval from the diagnosis of metastatic disease to thoracic RT was 8 months. Following thoracic RT, 26 patients (46%) achieved complete or partial response (overall response rate, ORR). Patients with squamous cell carcinoma or poorly-differentiated carcinoma had a higher ORR than those with adenocarcinoma (63% vs. 34%, *P* = 0.034). EGFR mutations was closely associated with a better ORR (45% vs. 29%, *P* = 0.284). At a median follow-up time of 44 months, the median OS, LRPFS after RT, and OS-RT were 50 months, 15 months, and 18 months.

**Conclusion:**

Radical palliative throractic RT is safe and might be beneficial for primary lung lesions of metastatic NSCLC patients with controlled extrathoracic diseases.

## Introduction

Clinically, approximately 50% of patients with non-small cell lung cancer (NSCLC) presented with metastatic disease at initial diagnosis; and the outcome of these patients is poor with a median overall survival of less than 1 year [1]. Uncontrolled distant metastasis accounts for a large proportion of cause of death in this subgroup of patients [2]. Systemic treatment and best supportive care are the main treatment modalities for metastatic NSCLC, while radiotherapy (RT) is primarily offerred in a palliative manner [1,3]. As the development of more effective systemic chemotherapy, including platinum-based combination and pemetrexed-based regimen, an increased proportion of long-term survival with metastatic NSCLC has been achieved [4–7]. In addition to systemic chemotherapy, for advanced NSCLC patients with epidermal growth factor receptor (EGFR) mutation, the use of EGFR–tyrosine kinase inhibitors (TKIs), such as gefitinib, erlotinib, or afatinib, as frontline or second-line treatment, can result in around 60% reduction in the risk of disease progression [8,9].

Once the metastatic disese is under controlled by systemic therapy, the effect of additional RT to the primary lung lesions is uncertain. There is increasing evidence that patients with stage IV disease and limited metastasis could benefit from aggressive thoracic RT beyond palliative intent [10–12]. Considering the additional thoracic RT may enhance local control, in clinical practice, we usually treat metastatic NSCLC patients who had controlled metastatic disease with aggressive RT for residual and progressive primary lung and mediastinal lesions in our institute.

We hypothesized that if the metastatic tumor volume of NSCLC is lowered by systemic treatment, radical thoracic RT might result in an increased survival through better local control, which in turn could prolong the effect of systemic treatment. In this study, we evaluated the clinicopathologic features, the RT dose, RT-related toxicities, and the clinical outcome of metastatic NSCLC patients who had controlled extrathoracic diseases after systemic treatment and subsequently received aggressive palliative thoracic RT. We also assessed the association between the RT response and histologic subtype (adenocarcinoma versus non-adenocarcinoma) and EGFR status (wild type versus mutation) of adenocarcinoma in these patients.

## Methods and Materials

### Patients

Between March 2006 and December 2014, 56 eligible patients were retrospectively reviewed and were included in this study. The diagnosis of NSCLC had to be cytologically or histologically confirmed. Karnofsky performance status scale was registered. At inclusion, all patients were subjected to the following diagnostic work-up: physical examination, radiologic examination (chest x-ray and computed tomography (CT) scan of the chest including liver and adrenal glands), cerebral computed tomography or magnetic resonance imaging, bone scan, and chemistry profile (cell counts, alkaline phosphatase, lactate dehydrogenase, gamma glutamyl transpeptidase, and serum creatinine). Systemic treatment with chemotherapy or molecular targeted therapy were given for metastatic NSCLC. If no progression of metastatic lesions, thoracic RT for stable or progressive primary lung lesions were given.

### Chemotherapy

The regimens and doses of the primary chemotherapy were adjusted individually according to the treatment response and side effects, and two-drug combinations were often used as first-line treatment options. Common regimens included platinum-based combination with new chemotherapy agents (gemcitabine, vinorelbine, paclitaxel, and docetaxel) and pemetrexed-based regimen [6, 13]. Targeted therapy with EGFR inhibitors such as gefitinib and erlotinib were also candidates as the 1^st^ or 2^nd^-line treatment [8,9].

### Thoracic Radiotherapy

Patients were immobilized with vacuum in the supine position with their both arms overhead. All paitients underwent CT-based simulation. The gross tumor volume (GTV) encompassed the primary lung tumor and/or gross mediastinal lymphadenopathies. If supraclavicular lymph nodes were involved, the GTV also contained these lesions. The clinical target volume (CTV) was defined as the GTV plus a 0.5 to 1 cm margin with regional mediastinal lymphatics. The planning target volume (PTV) was the CTV plus a margin of 0.5 cm to 0.8 cm for set-up uncertainty and respiratoy motion. The treatment planning were designed using three-dimensional conformal radiation therapy (3D-CRT) or intensity-modulated radiotherapy (IMRT) with 6 or 10 MV photon beams.

The radiotherapy was given in 1.8- to 2.5-Gy fractions, 5 days a week to a total dose of 40 Gy to 60 Gy according to the patient’s performance status and the tolerance of the adjacent normal organs. The mean lung dose, the percentage of the total lung volume receiving 20 Gy (V20), the maximal point dose of the spinal cord, the maximal point dose of the esophagus, and the mean esophagus dose were calculated [14].

### Evaluation of Treatment Response and Toxicity

Follow-up for thoracic tumor was performed with contrast-enhanced chest CT, generally 1–3 months after completing RT and at intervals of 3 to 6 months to assess for response or recurrence. All patients were evaluated according to Response Evaluation Criteria in Solid Tumrs (RECIST) criteria. RT-related acute toxicity was scored according to the National Cancer Institute’s Common Terminology Criteria for Adverse Events (CTCAE) 4.0 [15].

### Statistical Analysis

Overall survival rate (OS) was defined as time from diagnosis of NSCLC until death or until the patient was censored at time of last follow-up. Locoregional progression-free survival (LRPFS) was defined from the end of thoracic RT to locoregional thoracic failure, recurrence, or any cause of death or the last day of follow-up. Survival rate calculated from radiotherapy (OS-RT) was defined from the end of thoracic RT to any cause of death or the last day of follow-up. Survvial rates were estimated using the Kaplan-Meier method. The chi-square test was used to compare radiation response rate between groups

### Ethics Statement

The study protocol was approved by the Research Ethical Committee of National Taiwan University Hospital (NTUH: 201510051RINA). The patients' medical data were anonymized prior to access and analysis. The institutional review board has waived the need for written informed consent from study subjects because all potentially patient-identifying information was removed prior to data analysis.

## Results

### Patient Characteristics

A total of 56 patients with metastatic NSCLC were included in this study. The patient and tumor characteristics are listed in **Table 1.** There were 37 men and 19 women with a median age of 62 years old (range, 41–87 years). Among them, 49 patients had stage IV disease at initial diagnosis. The other 7 patients initially presented with localized disease and received lobectomy and lymph node dissection, but subsequently experienced recurrence with metastatic disease. More than 60% of all patients had T3/T4 disease, and the proportion of N2/N3 disease was also up to 64%. Among 32 patients with adenocarcinoma, 11 patients had activating mutations in the tyrosine kinase domain of the epidermal growth factor receptor (EGFR), while 10 patiens were wild-type and 11 patients had unknown status.

**Table 1:** Clinicopathologic features of total patients.

Characteristics		No. (%)
Age (years)	<60	29 (52)
	> = 60	27 (48)
Sex	Male	37 (66)
	Female	19 (34)
KPS	70%	3 (5)
	80%	9 (16)
	90%	37 (66)
	100%	7 (13)
Pathology	Adenocarcinoma	32 (57)
	Squamous cell carcinoma	16 (29)
	Poorly differentiated carcinoma	6 (10)
	Adenosquamous carcinoma	1 (2)
	Pleomorphic carcinoma	1 (2)
T-stage	T1	5 (9)
	T2	13 (23)
	T3	15 (27)
	T4	23 (41)
N-stage	N0	11 (20)
	N1	9 (16)
	N2	17 (30)
	N3	19 (34)
Local stage (ignoring M1 status)	I	6 (11)
	II	4 (7)
	IIIA	14 (25)
	IIIB	32 (57)
Localization metastasis	Bone	14 (20)
	Brain	8 (14)
	Lung	14 (25)
	Pleura	10 (18)
	Adrenal gland	3 (5)
	Liver	2 (4)
	Intra-abdominal lymph nodes	1 (2)
	skin	1 (2)

### Radiotherapy

The median dose of thoracic RT was 55 Gy (range, 40–62 Gy) delivered in 28 fractions (range, 20–30 fractions). The mean radiation dose to lung ranged from 4 Gy to 19 Gy (median, 12 Gy), and the median absolute lung volume treated above 20 Gy was 20% (V20, range: 9–34%). The maximal dose of spinal cord ranged from 2 to 49 Gy (median, 41 Gy). The maximal esophageal point dose ranged from 17 to 66 Gy (median, 54 Gy), and the mean dose of the whole esophagus ranged from 1 to 43 Gy (median: 17 Gy). The majority of the patients receivied additional palliative RT to extrathoracic regions before, after, and/or concurrent with thoracic RT (**Table 2**).

**Table 2:** Palliative radiotherapy to extrathoracic regions before, after, and/or concurrent with thoracic radiotherapy.

Timing and location of radiotherapy			No. (%)
Before pulmonary irradiation			11 (20)
	Bone		1
	Brain		8
	Spine		1
	Adrenal		0
	Others (SCF LN)		1
Concurrent with pulmonary irradiation			9 (16)
	Bone		2
	Brain		2
	Spine		3
	Adrenal		1
	Others (chest wall)		1
After pulmonary irradiation			31 (55)
	Bone		5
	Brain		12
	Spine		8
	Adrenal		2
	Others		4
		Soft tissue	2
		Mediastinal lymphadenopathy	1
		lung	1

**Abbreviation:** SCF, supraclavicular fossa; LN, lymph node.

### Chemotherapy

Forty-eight (86%) received at least one couse of chemotherapy alternating with or without molecular targeted therapy, and 10 patients (18%) received molecular targeted therapy only as their primary treatment. Twenty-nine (52%) patiens had concurrent chemotherapy during thoracic RT, and 41% of them had two-drugs combinations (**Table 3**).

**Table 3:** Treatment for primary tumor and lymph nodes.

Treatment		No. (%)
RT alone		27 (48)
Concurrent chemoradiotherapy		29 (52)
	Cisplatin-etoposide	5
	Cisplatin-pemetrexed	2
	Cisplatin-docetaxel	5
	Vinorelbine	1
	Gefitinib	8
	Pemetrexed	3
	Cetuximab	1
	Paclitaxel	1
	Docetaxel	3
Radiotherapy dose (median/range)		55 Gy / 40Gy-60 Gy
Fraction size (median/range)		2 Gy / 1.8 Gy-2.5 Gy
Overall treatment time of radiotherapy (median/range)		37 days/ 25–47 days

**Fig 1:**
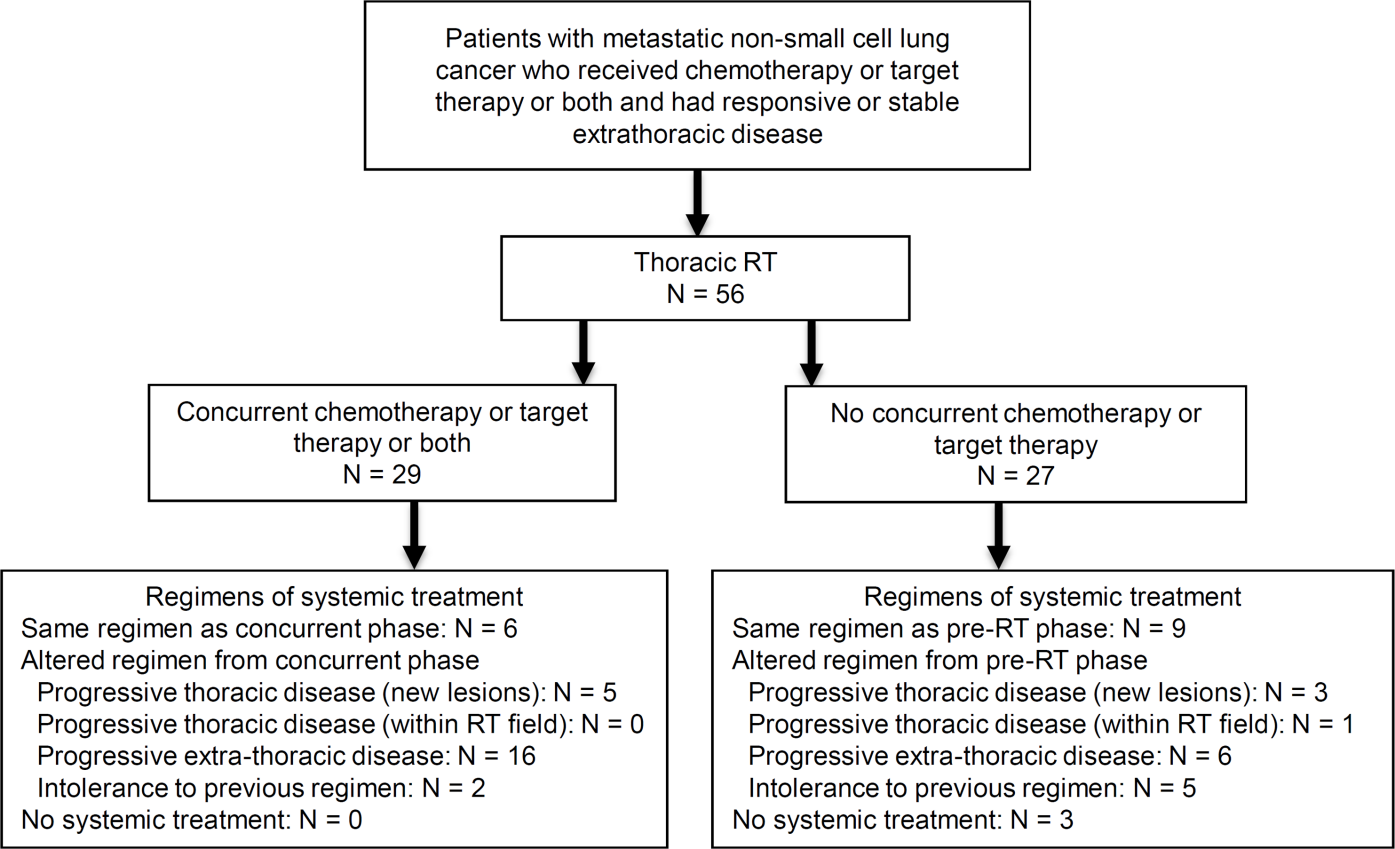
Schema of 56 NSCLC patients received aggressive palliative thoracic RT with and without systemic treatment. Numbers and proportions of patients with or without altered systemic therapy regimens after thoracic RT depending on the treatment response and clinical adjustment. RT, radiotherapy; N, number.

Among the 27 patients receiving thoracic RT without concurrent systemic treatment, 9 patients (33.3%) received the same systemic therapy regimen as that of the prethoracic RT, whereas 15 (55.6%) patients received alternative regimens that were different from those of the prethoracic RT because of progressive disease (PD) after RT and intolerance to the previous regimen (**Fig 1**). Moreover, 3 patients (11%) who discontinued systemic therapy after RT achieved a stable disease condition at 4, 5, and 10 months, respectively, until the latest follow-up. Among the 29 patients who had undergone concurrent chemoradiotherapy (CCRT) or received molecular target agents, 6 (20.7%) were maintained on the same pre-RT medication after RT, whereas the remaining 23 (79.3%) patients were treated with different systemic regimens because of PD and intolerance to the pre-RT regimens.

Overall, 15 patients with thoracic RT alone and 23 patients with CCRT or molecular target agents received altered regimens of systemic therapy that were different from the pre-RT regimen. The median interval to shift of the different systemic therapy regimens after completing thoracic RT was 5 months (range, 6 days to 31 months). The clinical decisions for changing medication for these 38 patients with thoracic RT or thoracic CCRT (including molecular target agents) were progressive extrathoracic (22/38, 58%) and thoracic diseases (9/38, 24%) after thoracic RT in addition to the intolerant side effects of the original regimens (7/38, 18%) (**Fig 1**).

### Treatment Response

**Fig 2:**
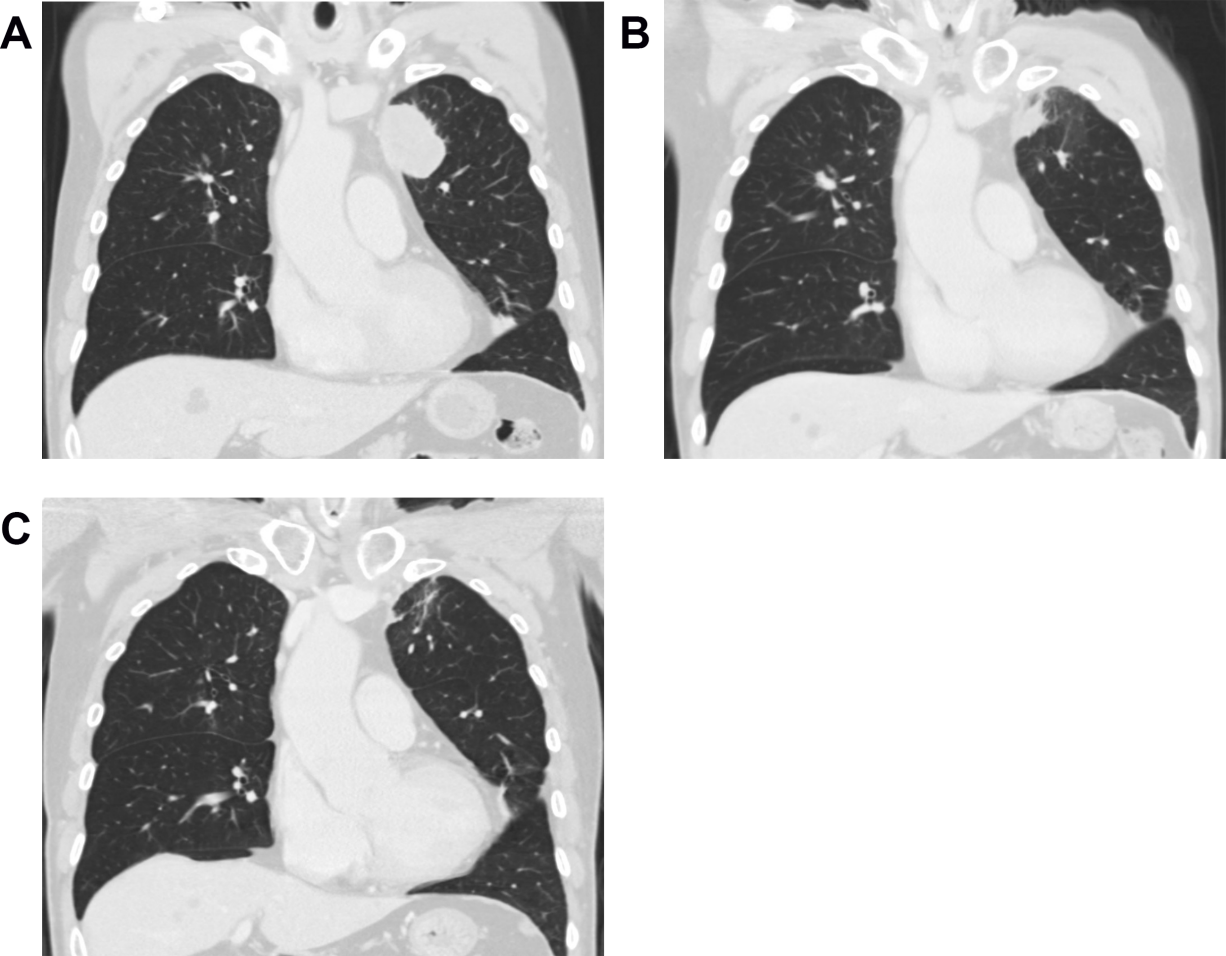
One patient presented with complete remission of thoracic tumor after radiotherapy. This 66-year-old man had initial lung to lung metastasis, and the disease was controlled by chemotherapy with docetaxel and cisplatin. However, tumors at left upper lobe and mediastinum progressed 5 months after diagnosis. Mediastinal lesions improved after chemotherapy with gemcitabine and vinorelbine, but left upper lung tumor remained stationary. Thoracic radiotherapy (RT) with 55 Gy in 25 fractions was applied to left lung tumor and 45 Gy in 25 fractions to mediastinal lymphatics using IMRT. Complete remission of thoracic lesions was achieved five months after completing RT. Only grade 1 radiation pneumonitis was noted. He remained disease-free 17 months after RT without systemic chemotherapy. (A) Chest CT scan before thoracic RT. (B) Chest CT scan two months after thoracic RT. (C) Chest CT scan five months after thoracic RT.

Twenty-six patients (46%) achieved complete response (CR) or partial response (PR), while 26 patients (46%) had stable disease (SD) and 4 patients (8%) had PD. As shown in **Fig 2**, one patient had CR of thoracic tumor 4 months after completing 55 Gy. He was alive without progression at the latest follow-up.

Patients with squamous cell carcinoma or poorly-differentiated carcinoma had a higher overall response rate (ORR, CR+PR) than those with adenocarcinoma (63% vs. 34%, *P* = 0.034). Among patients with adenocaricnoma, those with EGFR mutations had a trend better ORR than those with wild-type and unknown status (45% vs. 29%, *P* = 0.284). There were no differences of chemotherapy regimen and radiation dose between adenocarcinoma and non-adenocarcinoma. Among patients with adenocarcinoma, the chemotherapy regimen and radiation dose were not different between mutant-EGFR and wild-type/unknown EGFR.

### Survival

**Fig 3:**
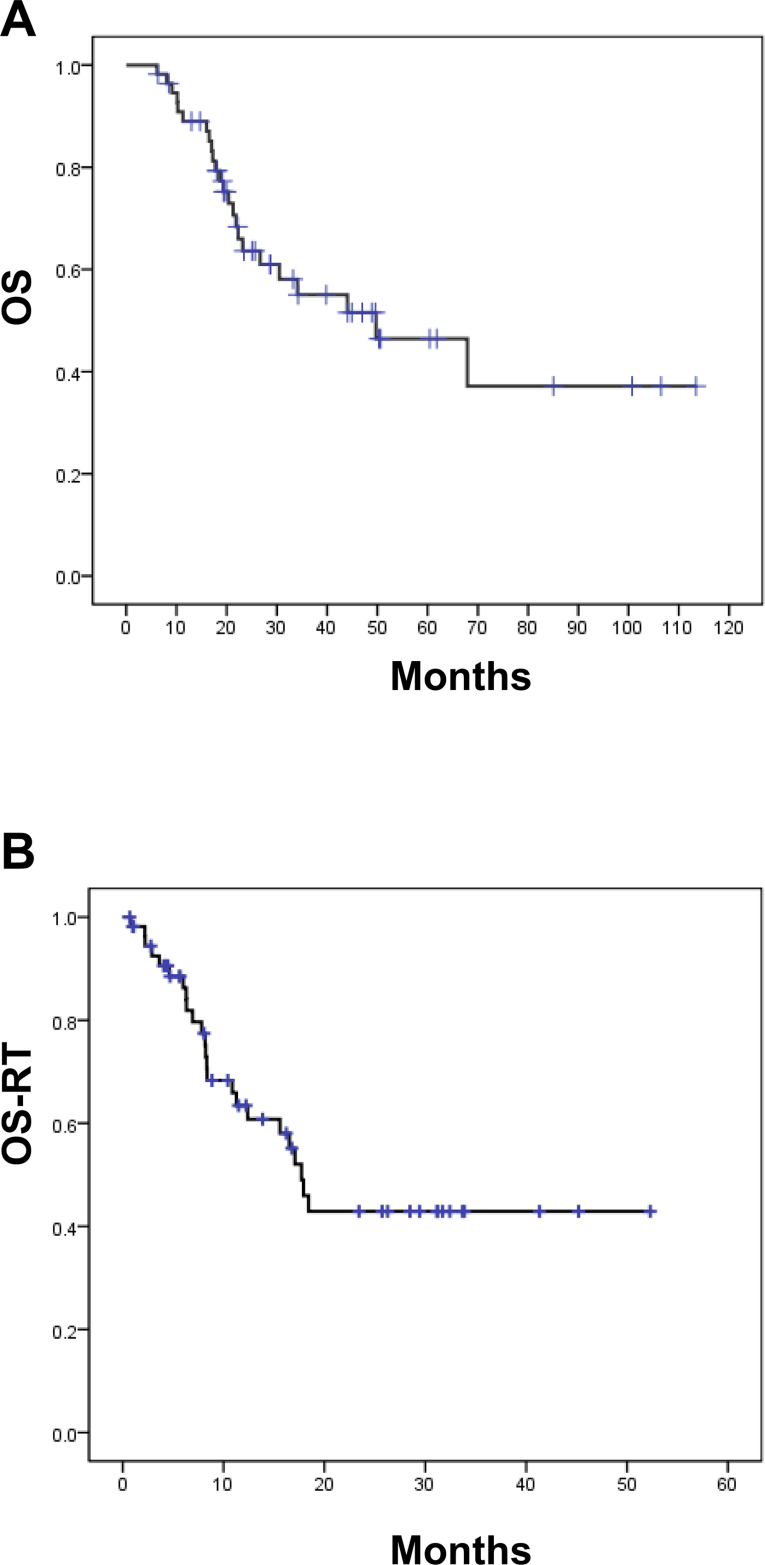
Survival rates of 56 patients receiving aggressive palliative thoracic RT. (A) Overall survival (OS) of all patients (B) Overall survival rate calculated from radiotherapy (OS-RT).

Thirty-five (63%) patients were alive after a median follow-up of 44 months (range: 6–113 months). Median interval from the diagnosis of metastatic disease to thoracic RT was 26 months (range, 1–81 months). Median OS (**Fig 3A**) and median OS-RT (**Fig 3B**) were 50 months and 18 months, respectively. The 5-year OS for total 56 patients was 43%, whereas 2-year OS-RT was 41%. The median and 2-year LRPFS after the completion of RT were 15 months and 33%, respectively, for 56 patients. Among the 27 patients who received thoracic RT alone, the median and 2-year LRPFS after the completion of RT were 12 months and 31%, respectively. Furthermore, the median and 2-year LRPFS after the completion of RT were 18 months and 36%, respectively, in patients receiving combined systemic therapy and thoracic RT

### Treatment-related Toxicity

Five patients (14%) experienced neutropenic fever, 3 patients (7%) experienced grade 2 toxicites of radiation pneumonitis, and 7 patients (16%) had acute grade 2 esophagitis, while there was no reported grade 3 or 4 toxicity for acute radiation pneumonitis and esophagitis (**Table 4**). Hematology toxicity was the most common and severe complication, which might be related to concurrent or induction chemotherapy. The majority of patients up to 80% had grade 1 or grade 2 late toxicity of radiation pneumonitis, but no grade 3 and 4 severe late toxicities occurred.

**Table 4:** Acute toxicity of thoracic radiotherapy.

Patient no. (%)	Grade 1	Grade 2	Grade 3	Grade 4
Fatigue	12 (21)	3(5)	0	0
Dysphagia	10 (22)	7 (16)	0	0
Dyspnea	8 (14)	0	0	0
Cough	13 (23)	1 (2)	0	0
Pneumonitis	10 (18)	6 (11)	0	0
Neutropenic fever	-	-	8 (14)	0
Dermatitis	9 (16)	5 (9)	1 (2)	0

## Discussion

Increasing evidences have demonstrated that maintenance chemotherapy can prolong the survival rate for advanced NSCLC patients with well controlled disease after administration of platinum-based combination regimen [16,17]. In addition to maintenance chemotherapy, prolonged use of molecular targeting agents decreases the progression and subsequently increases the survival for advanced NSCLC patients with EGFR mutation [8,9]. However, a great proportion of patients died of intrathoracic disease, even if metastatic lesions have been well-controlled after systemic treatment. Another reason is that systemic therapy became ineffective for primary thoracic lesions. On the other hand, for patients with controlled extrathoracic disease, if intrathoracic disease is effectively controlled by local treatment, the survival can be prolonged.

In this study, our results demonstrate that in patients with metastatic NSCLC with controlled extrathoracic disease, radical thoracic RT with a median dose of 55 Gy resulted in an ORR of 46% and 2-year LRPFS and OS of 27% and 41%, respectively, after the completion of RT. Furthermore, in patients who received thoracic RT alone, RT with a median dose of 55 Gy resulted in an ORR of 52%, and 2-year LRPFS and OS of 31% and 41%, respectively, after the completion of RT. Of these patients, 15 received the previous systemic regimen, and 3 did not receive further systemic treatment; however, their condition remained stable for 4–10 months. These findings suggest that radical thoracic RT alone can improve and enhance local control of thoracic lesions if the extrathoracic disease is well controlled by systemic therapy. The possibilities of the better local control and the subsequent benefit of survival of our patients receiving radical thoracic RT are: (1) If stationary or progressive primary lung lesions can be controlled by RT, the physician could continue using current systemic medications, which were effective to other metastatic lesions, rather than switching to another regimen at the risk of losing systemic control. (2) The use of modern RT techniques, such as IMRT [18], can not only increase the homogenous dose to gross tumor but also diminish RT-related adverse effects, such as pneumonitis and esophagitis, and thus lessen the morbidities.

Palliative-intent RT is effective for improvement of intrathoracic disease-related symptoms, such us cough, chest pain, dyspnea and airway obstruction [19,20]. However, the optimal dose of thoracic RT for symptom relief and local control for primary lesions has not been well defined. Even more controversial is what impact of palliative RT on survival for metastatic NSCLC patients [21,22]. In a systemic review of randomized-controlled trials with palliative thoracic RT for advanced NSCLC, Fairchild et al [23] reported that RT with more than 35 Gy_10_ biologically equivalent dose (BED) (higher-dose RT arm) causes a greater symptom improvement (improved total symptom score, 77.1% patients versus 65.4% patients, *P* = 0.003) and a better one-year survival (26.5% versus 21.7%, *P* = 0.002) than lower BED arm. However, higher-dose RT arm was significantly associated with greater physician-assessed dysphagia when compared with lower-dose RT arm (20.5% versus 14.9%, *P* = 0.01) [23]. The authors suggest that higher-dose thoracic RT improves symptoms, such as hemoptysis, obstructive pneumonitis, and performance status that allows patients have opportunities to receive further systemic treatment and thus benefit from the prolonged survival [23]. Along this line, our current study demonstrated that higher-dose thoracic RT (median dose of 55 Gy was more than 35 Gy_10_ BED) resulted in a greater survival (one-year OS, 88%), and a less toxicity of radiation esophagitis (grade 2, 16%).

In the present study, the thoracic RT dose is dependent on the normal organ constraints, such as mean lung doses were all less than 20 Gy, the majority of V20 was less than 32% (89% patients) [24,25], and the mean esophageal dose of most patients (75% patients) was less than 28 Gy [26]. This RT treatment strategy may explain that our patients had less toxicity of radiation pneumonitis and radiation esophagitis, even though a median thoracic RT dose is around 55 Gy. This approach may also allow patients to continuously receive systemic chemotherapy or molecular target agens, and this treatment modality produces a better 2-year OS of 41% after completing RT when compared with historical data concerning patients who had been treated with third-generation chemotherapy regimens or newer agents [27–29]. Our findings are also in line with a recent observation that the concurrent chemotherapy and thoracic RT resulted in a median survival of 10.0 months, and the 2-year OS rates of 16.4% for stage IV NSCLC patients, although the median RT dose for primary tumor is 63 Gy [30].

Several studies have also demonstrated that patients of stage IV NSCLC with oligometastasis can benefit survival from radical treatment to all macroscopic tumor sites, including primary thoracic gross lesions [10–12,31,32]. These findings suggest that once metastatic tumor volume is reduced by systemic chemotherapy, additional radical thoracic RT can improve survival rate by enhancing local tumor control. Notably, our study showed that patients with squamous cell carcinoma or poorly-differentiated carcinoma had a higher ORR than those with adenocarcinoma. This finding indicate that patients with squamous cell carcinoma or poorly-differentiated carcinoma who progrssed after systemic chemotherapy still have a change to improve survival after receiving thoracic RT. Our results also showed the close association between a higher overall response rate and EGFR mutation among adenocarcinoma. This result further supported our previous finding that among adenocarcinoma patients, EGFR mutation (*P* = 0.029) was independently associated with response to whole brain radiotherapy [33].

Although we can’t exclude the selection bias in this study, for example, patients with controlled extrathoracic metastatic lesions relatively had better good performance status and tolerated higher-RT dose, our results reveal that radical palliative thoracic RT is safe and might be beneficial for metastatic NSCLC with controlled extrathoraic disease. This crucial finding indicates that aggressive palliative thoracic RT can improve the survival time for this group of patients if distant metastatic lesions of NSCLC are well controlled using chemotherapy or molecular target agents alone or in combination. For NSCLC patients with squamous cell carcinoma or poorly-differentiated carcinoma histologic subtype who are comparatively respond to RT, the escalation of RT dose to increase the response rate and thus to recuperate the survival is suggested. Further prospective studies to clarify the optimal RT dose and to evaluate the benefit of the additing radiosensitizers during RT for this subgroup of patients are warranted.
